# Does Perioperative Subcutaneous Heparin or Intravenous Tranexamic Acid Affect the Rate of Vascular Complications in Anterior Lumbar Interbody Fusion Procedures?

**DOI:** 10.7759/cureus.81429

**Published:** 2025-03-29

**Authors:** Michael Albdewi, Mark M Zaki, Kelsey Fearer, Rushikesh S Joshi, Jacob R Joseph, Rakesh D Patel, Osama Kashlan

**Affiliations:** 1 Neurosurgery, University of Michigan, Ann Arbor, USA; 2 Orthopaedic Surgery, University of Michigan, Ann Arbor, USA; 3 Neurosurgery, Weill Cornell Medicine, New York, USA

**Keywords:** anterior lumbar interbody fusion, heparin, prophylaxis, thrombosis, tranexamic acid

## Abstract

Introduction

Anterior lumbar interbody fusion (ALIF) is a surgical technique commonly used to treat degenerative disk disease in the lower lumbar spine. As the procedure commonly involves retraction of the iliac arteries and veins, potential complications include deep vein thrombosis (DVT) and hemorrhagic vessel injuries. The goal of this retrospective review is to assess whether the use of intraoperative intravenous tranexamic acid (TXA), subcutaneous heparin (SCH), or neither is associated with clinical complications and surgical outcomes.

Methods

All patients undergoing ALIF from 2015 to 2023 at a tertiary academic medical center were reviewed. Intraoperative use of prothrombotic or anticoagulant and short- and long-term outcomes were assessed.

Results

One hundred seventy-nine patients were included; there were 81 (45.3%) female patients and an average age of 58.96 ± 13.34 years. Twenty-eight patients received SCH, 34 patients received TXA, and 117 received neither. The use of perioperative SCH or TXA in ALIF procedures did not result in statistically significant differences in complication rates or pain scores. Statistically significant differences were found with the use of coagulative intervention and blood transfusions, with the TXA requiring the fewest transfusions and the no-intervention group requiring the most. Patients receiving TXA were more likely to be discharged home without the need for any other services. Although not statistically significant, there was a trend of decreasing estimated blood loss (EBL) between the coagulation intervention groups, with no intervention having the highest EBL, followed by SCH and TXA.

Conclusion

The use of perioperative coagulative intervention in ALIF procedures did not result in a significant change in complication rates, hospital stays, or pain outcomes. The TXA group had a trend toward lower blood loss, no patients requiring a blood transfusion, a higher likelihood of being discharged home without any supplemental services, and the best neurologic outcomes. As such, further large and prospective trials should be performed to study the effect of TXA in ALIF patients further to determine whether patients undergoing ALIFs would benefit from TXA administration.

## Introduction

Anterior lumbar interbody fusion (ALIF) stands as a cornerstone surgical technique for managing degenerative disk disease, particularly in the lower lumbar spine and lumbosacral junction [1]. Distinguished from posterior lumbar interbody fusion (PLIF), the ALIF approach minimizes posterior muscle dissection, reduces neuronal element exposure, and mitigates the risk of injury [2]. Additionally, it facilitates the placement of larger interbody cages, thereby enhancing lordosis and promoting indirect foraminal decompression [2]. Between 2007 and 2014 in the United States, the number of ALIF procedures increased significantly, with a yearly growth rate of 24%, rising from 3,650 to 6,151 procedures [3]. With the rise in life expectancy, the demand for ALIF procedures among adults seeking treatment for degenerative disk disease is expected to continue increasing.

Despite its manifold advantages, ALIF procedures necessitate the involvement of general or vascular surgeons for operative exposure and pose its own unique complications [4]. Complications associated with anterior exposure involve vascular problems, such as vessel injury or thrombosis [2,5]. Other common postoperative complications include urinary tract infections (UTIs), ileus, retrograde ejaculation, hernia, peritoneal entry, deep vein thrombosis (DVT), and pulmonary embolism [6]. Intraoperatively, ALIF procedures often require the retraction of the iliac vein, which may increase the risk of thrombotic events.

There is heterogeneity in the use of perioperative coagulative interventions to reduce the risk of thrombotic and hemorrhagic events during ALIF procedures. Use of anticoagulation during ALIF procedures may reduce the thrombotic risk posed by prolonged retraction of the iliac vein; however, antifibrinolytic interventions may also increase the risk of severe bleeding events in the setting of vessel injury. At our institution, there is a spectrum of practices regarding perioperative coagulative intervention for ALIF procedures, ranging from subcutaneous heparin (SCH) and tranexamic acid (TXA) to neither. We, thus, sought to determine if the use of perioperative SCH, TXA, or neither is associated with an increased risk of clinical complications and surgical outcomes.

## Materials and methods

A retrospective, single-institution review of medical records of patients who had undergone ALIF at the University of Michigan from January 2015 through February 2023 was performed. Patient demographic and clinical information was collected including age, sex, race, ethnicity, baseline anticoagulant use prior to surgery, length of surgery, estimated blood loss (EBL), surgical approach (anterior; anterior and lateral; anterior and posterior; and anterior, lateral, and posterior), intraoperative coagulative intervention (no intervention, TXA, or SCH), complications (hemorrhage, DVT, leg swelling, calf pain, shortness of breath, O_2_ requirement, pulmonary embolism, myocardial infarction, UTI, surgical site infections (SSIs), hematoma, cerebral vascular accident (CVA), durotomy, pneumonia, and ileus), transfusions, surgeon specialty (neurosurgeon or orthopedic surgeon), and surgeon experience. Patients were excluded if they did not undergo surgery at the University of Michigan during the specified period or did not undergo an anterior approach for spinal fusion. The standard dosing of SCH and TXA was 5,000 U and 1 g, respectively, and was given during patient induction. Additionally, information on outcomes including discharge disposition, discharge location, and change in visual analog scale (VAS) pain scores and neurologic deficits at presentation and one- to three-month follow-ups were recorded. Due to missing chart information, only 154 patients were included in VAS analyses.

All statistical analyses were performed controlling for age, sex, ethnicity, race, and whether a vessel injury occurred during the surgery. Patients were divided into three groups based on coagulative intervention: no intervention, SCH, or TXA. Treatment group assignment was based on surgeon preference. Operative complications between the three coagulative intervention groups were compared with binary logistic regression. The Hosmer-Lemeshow test indicates that the model provided a good fit for the data (see the appendix).

EBL between the three coagulative intervention groups was compared with linear regression. Analysis between operative complications did not have vessel injury controlled for, as this was one of the complications. Change in preoperative and postoperative motor function between the coagulation groups was compared via chi-squared analysis. A pairwise analysis was included in the analysis of surgical length. Differences in discharge location and blood transfusion requirement were compared between the coagulation groups via chi-squared analysis. Change in preoperative and postoperative VAS pain scores between the coagulation groups was compared via ANOVA. All statistical analysis was performed using the R Programming Language via RStudio Version 2022.12.0 Build 353 for Windows (Posit, Boston, MA, US) and IBM SPSS Statistics Version 29.0.0.0 (241) (IBM Corp., Armonk, NY, US). The Institutional Review Board of the University of Michigan waived the need for ethics approval and the need to obtain consent for the collection, analysis, and publication of the retrospectively obtained and anonymized data for this non-interventional study (IRB#: HUM00213958).

## Results

One hundred seventy-nine patients were included in this study; there were 81 female patients (45.3%) and an average age of 58.96 ± 13.34 years. After dividing the patients by coagulative intervention, there were 117 patients (65.3%) who received no intervention, 28 patients (15.6%) who received SCH, and 34 patients (19.0%) who received TXA (Table 1). There was a statistically significant difference in the average age of patients in the no intervention, SCH, and TXA, which were 60.63 ± 13.19, 60.46 ± 11.79, and 51.94 ± 13.28 years, respectively (χ^2^ = 10.6, p = 0.01). Age was controlled for in the statistical analysis. There was no statistically significant difference in the distribution of sex with 52 (44.4%), 13 (46.4%), and 16 (47.1%) female patients in the no-intervention, SCH, and TXA groups, respectively (χ^2^ = 0.09, p = 0.955). There were no significant differences in baseline anticoagulant use prior to surgery (p = 0.073, χ^2^ = 5.24) between the three coagulative intervention groups. There was a statistically significant difference in the anterior-only surgical approach between the three coagulative intervention groups with zero, five (17.9%), and 23 (19.7%) patients in the TXA, SCH, and no-intervention groups undergoing this approach, respectively (χ^2^ = 7.84, p = 0.02). Additionally, there was a statistically significant difference in the combined anterior and posterior surgical approach between the three coagulative intervention groups with 30 (88.2%), 20 (71.4%), and 78 (66.7%) patients in the TXA, SCH, and no-intervention groups undergoing this approach, respectively (χ^2^ = 6.02, p = 0.05). There were no differences in the combined anterior and lateral or combined anterior, lateral, and posterior approaches (Table 1).

**Table 1:** Patient Demographics and Coagulative Interventions This table summarizes the baseline characteristics of the patients included in the study with divisions by coagulative intervention strategies. NI: no intervention; SCH: subcutaneous heparin; TXA: tranexamic acid

	Overall (n = 179)	NI (n = 117)	SCH (n = 28)	TXA (n = 34)	p-value (chi-squared value)
Average age (±SD)	59 ± 13	61 ± 13	60 ± 12	52 ± 13	0.01 (χ^2^ = 10.6)
Sex					0.955 (χ^2^ = 0.09)
Male (n, %)	98 (54.8%)	65 (55.6%)	15 (53.6%)	18 (52.9%)	
Female (n, %)	81 (45.3%)	52 (44.4%)	13 (42.4%)	16 (47.1%)	
Surgical approach					
Anterior (n, %)	28 (15.6%)	23 (19.7%)	5 (17.9%)	0	0.02 (χ^2^ = 7.84)
Anterior and lateral (n, %)	2 (1.1%)	1 (0.9%)	1 (3.6%)	0	0.37 (χ^2^ = 1.98)
Anterior and posterior (n, %)	128 (71.5%)	78 (66.7%)	20 (71.4%)	30 (88.2%)	0.05 (χ^2^ = 6.02)
Anterior, lateral, and posterior (n, %)	18 (10.1%)	13 (11.1%)	1 (3.6%)	4 (11.8%)	0.46 (χ^2^ = 1.56)
Baseline anticoagulation use (n, %)	50 (27.9%)	39 (33.3%)	6 (21.4%)	5 (13.9%)	0.073 (χ^2^ = 5.24)

Forty-two (23.5%) complications were found in the cohort: 28 (23.9%) from the no-intervention group, eight (28.6%) from the SCH group, and six (17.6%) from the TXA group. The most common complications were calf pain and ileus, with six and 17 patients from the entire cohort with complications experiencing each, respectively. There was no statistically significant association between any complication and coagulative intervention (Table 2).

**Table 2:** Incidence of Perioperative Complications Among Coagulative Intervention Groups This table details the frequency of postoperative complications, comparing patients who received SCH, TXA, or no intervention. Each row represents a specific complication with three p-values, one for each coagulative intervention. Each p-value corresponds to statistical comparisons of that complication’s incidence between each intervention group and the two other groups (i.e., NI vs. others, SCH vs. others, and TXA vs. others). Binary logistic regression was used for statistical analysis; validation of the model may be found in the appendix. ^1^Defined as PE and DVT NI: no intervention; SCH: subcutaneous heparin; TXA: tranexamic acid; CVA: cerebral vascular accident; DVT: deep vein thrombosis; PE: pulmonary embolism; SOB: shortness of breath; SSI: surgical site infection; UTI: urinary tract infection

Complication	NI (n = 117)	SCH (n = 28)	TXA (n = 34)	p-values
DVT	1 (0.85%)	0	0	NI: 1.000
				SCH: 0.999
				TXA: 0.999
Leg swelling	2 (1.71%)	2 (7.14%)	0	NI: 0.481
				SCH: 0.226
				TXA: 0.998
Calf pain	4 (3.42%)	2 (7.14%)	0	NI: 0.339
				SCH: 0.141
				TXA: 0.998
SOB	3 (2.56%)	0	1 (2.94%)	NI: 0.976
				SCH: 0.998
				TXA: 0.824
PE	1 (0.85%)	0	0	NI: 1.000
				SCH: 0.998
				TXA: 0.998
Thrombotic events^1^	2 (1.71%)	0	0	NI: 1.000
				SCH: 0.998
				TXA: 0.998
Vessel injuries	1 (0.85%)	0	0	NI: 1.000
				SCH: 0.998
				TXA: 0.998
UTI	0	2 (7.14%)	1 (2.94%)	NI: 0.769
				SCH: 0.995
				TXA: 0.995
SSI	1 (0.85%)	0	0	NI: 1.000
				SCH: 0.998
				TXA: 0.998
Hematoma	1 (0.85%)	0	0	NI: 1.000
				SCH: 0.998
				TXA: 0.998
CVA	2 (1.71%)	0	0	NI: 1.000
				SCH: 0.998
				TXA: 0.998
Durotomy	0	1 (3.57%)	0	NI: 1.000
				SCH: 0.996
				TXA: 1.000
Ileus	12 (10.26%)	1 (3.57%)	4 (11.76%)	NI: 0.437
				SCH: 0.369
				TXA: 0.445

There was no statistically significant difference between the change in motor function, either improvement or worsening, and coagulative intervention (χ^2^ = 12.55, p = 0.0508; Table 3).

**Table 3:** Changes in Postoperative Motor Function Across Coagulative Intervention Groups This table compares the preoperative and postoperative motor function of patients to assess whether different coagulative strategies influenced neurological outcomes. Chi-squared analysis was performed to determine statistical significance (χ^2^ = 12.55, p = 0.0508). NI: no intervention; SCH: subcutaneous heparin; TXA: tranexamic acid

Motor outcome	NI (n = 117)	SCH (n = 28)	TXA (n = 34)	p-value (chi-squared value)
Decreased postop function	4 (3.42%)	4 (14.29%)	0	0.0508 (χ^2^ = 12.55)
No change - with pre-existing deficit	13 (11.11%)	2 (7.14%)	1 (2.94%)	
No change - without pre-existing deficit	79 (67.52%)	20 (71.43%)	25 (73.53%)	
Improved postop function	21 (17.95%)	2 (7.14%)	8 (23.53%)	

The average change in preoperative to postoperative VAS pain scores in the cohort was -2.17 ± 3.24. There was no statistically significant difference in change in VAS pain scores between the different coagulation intervention groups (F = 1.286, p = 0.322; Table 4).

**Table 4:** Comparison of Preoperative to Postoperative VAS Pain Scores This table presents changes in patient-reported pain scores following spinal fusion, stratified by the coagulative intervention group. ANOVA was used for statistical analysis (F = 1.286, p = 0.322). NI: no intervention; SCH: subcutaneous heparin; TXA: tranexamic acid; VAS: visual analog scale

Coagulative intervention	Average VAS change	p-value (F-value)
NI (n = 117)	-1.93 ± 3.17	0.322 (1.286)
SCH (n = 28)	-2.58 ± 3.26	
TXA (n = 34)	-2.72 ± 3.35	

Twenty-seven patients required blood transfusions during or shortly after their operations. There was a significant difference in the transfusion rates between the coagulative intervention groups with 24 (20.5%) patients in the no-intervention group, three (10.7%) in the SCH group, and zero patients in the TXA group requiring transfusions (p = 0.034; Table 5).

**Table 5:** Blood Transfusion Rates in Patients Undergoing Spinal Fusion Across Coagulative Intervention Groups This table highlights the number of patients requiring blood transfusions based on the coagulative intervention received. Chi-squared analysis was used to determine statistical significance (χ^2^ = 7.72, p = 0.034). NI: no intervention; SCH: subcutaneous heparin; TXA: tranexamic acid

	No blood transfusion	Blood transfusion	p-value (chi-squared value)
NI (n = 117)	93 (79.5%)	24 (20.5%)	0.034 (χ^2^ = 7.72)
SCH (n = 28)	25 (89.3%)	3 (10.7%)	
TXA (n = 34)	34 (100%)	0	

**Figure 1:**
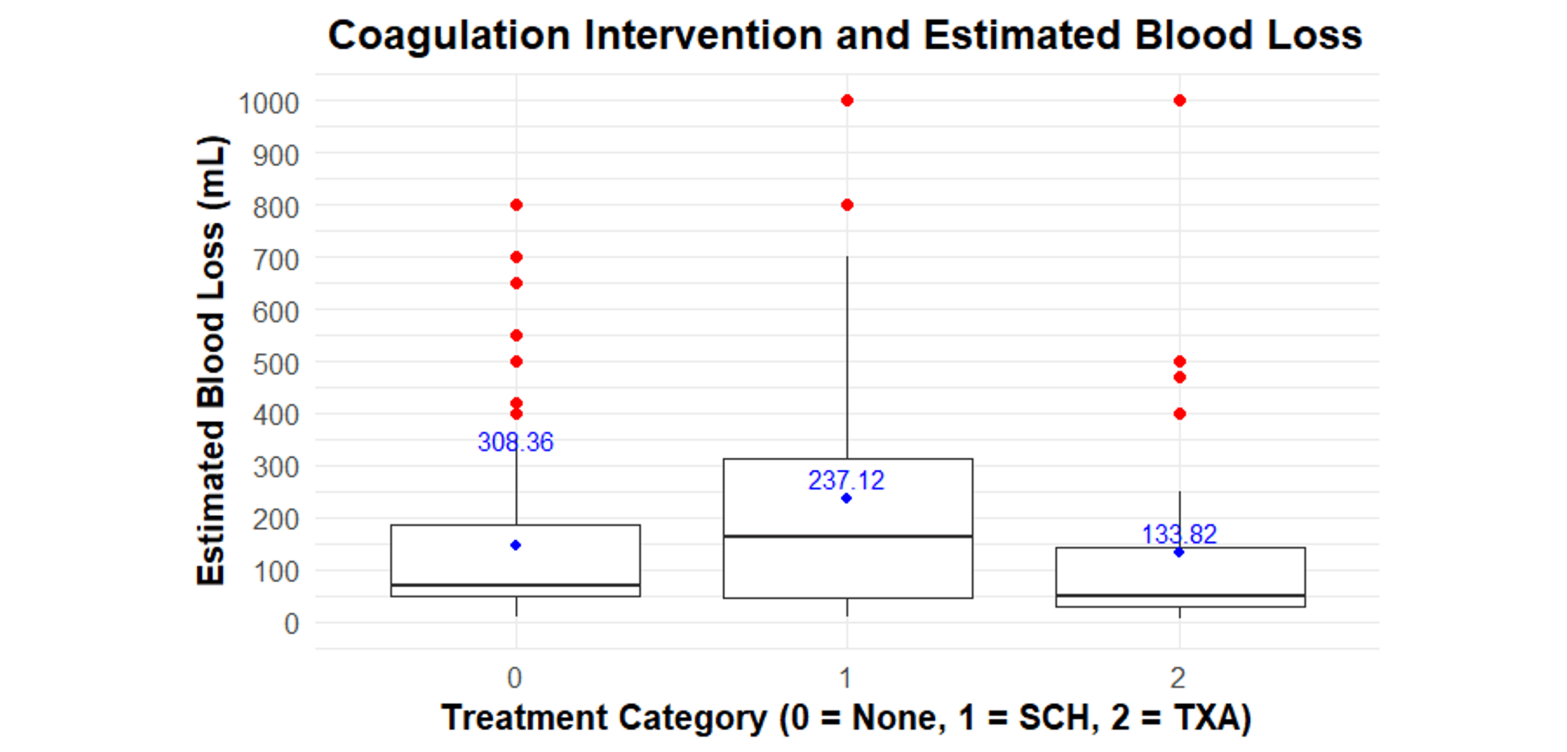
Estimated Intraoperative Blood Loss by Coagulation Intervention Group This figure illustrates the mean EBL with standard deviations for each coagulative intervention group. Linear regression was used to determine statistical significance (p = 0.192). EBL: estimated blood loss; SCH: subcutaneous heparin; TXA: tranexamic acid

Within the entire cohort, there was no statistically significant association between EBL and coagulative intervention (p = 0.192). However, though not statistically significant, the TXA group had the lowest EBL (Figure 1).

There was no statistically significant decrease in the average length of surgery between the TXA group, SCH group, and no-intervention group (p = 0.74; Table 6).

**Table 6:** Comparison of Average Surgical Times Among Coagulative Intervention Groups This table displays the duration of ALIF procedures, analyzing the differences in surgical time between coagulative intervention groups. Multiple linear regression was performed to evaluate for statistical significance (p = 0.74 for coagulative intervention, p = <0.001 for the model). NI: no intervention; SCH: subcutaneous heparin; TXA: tranexamic acid; ALIF: anterior lumbar interbody fusion

Coagulative intervention	Average length of surgery (minutes)	p-value
NI (n = 117)	274.9 ± 125.3	0.74
SCH (n = 28)	339.3 ± 154.2	
TXA (n = 34)	352.3 ± 130.7	

There was a significant difference between discharge location and coagulation intervention, with TXA being associated with an increased probability of being discharged home (χ^2^ = 18.97, p < 0.001; Table 7).

**Table 7:** Discharge Disposition of Patients Undergoing ALIF Based on Coagulative Intervention Groups This table examines patient discharge locations, including routine home discharge, home with additional healthcare services, or transfer to post-acute care facilities. Chi-squared analysis was used to determine statistical significance (χ^2^ = 18.97; p < 0.001). NI: no intervention; SCH: subcutaneous heparin; TXA: tranexamic acid

	Home routine	Home routine with healthcare services	Post-acute/non-acute care setting	p-value (chi-squared value)
NI (n = 117)	71 (60.7%)	20 (17.1%)	26 (22.2I%)	<0.001 (χ^2^ = 18.97)
SCH (n = 28)	18 (64.3%)	4 (14.3%)	6 (21.4%)	
TXA (n = 34)	34 (100%)	0	0	

## Discussion

ALIF procedures are widely used and effective for treating degenerative disk disease, particularly due to their strong lordosis correction capabilities. The increasing utilization and related complications of ALIF highlight the need for further research into perioperative management to establish clinical standards and improve medical outcomes. Given the heightened risks of DVT and PE associated with ALIF, it is crucial to understand how modifiable factors, such as coagulative interventions, impact patient outcomes to reduce surgical complications [3,6]. In this study, we aimed to determine whether variations in perioperative coagulation management were linked to complications, surgical metrics, or clinical outcomes. Our findings did not show any significant associations with complications, clinical outcomes, or most surgical metrics. However, although no statistically significant difference in EBL was found among coagulative intervention groups, a trend of decreasing blood loss was noted from the no-intervention group to the SCH group and further to the TXA group. Furthermore, blood transfusions and discharge locations were associated with coagulation intervention.

Previous studies have primarily compared the effects of intraoperative heparin administration with no administration [7,8], whereas our study also included a comparison to TXA administration. Claydon et al. [7] found that heparinized patients undergoing anterior lumbar surgery had significantly higher blood loss (104 mL) compared to the non-heparin group (53 mL). Similarly, Sim et al. [8] reported that heparinized patients experienced greater blood loss (389.7 mL) than unheparinized patients (160.5 mL). In contrast to these findings, our study did not find a significant difference in EBL among the heparinized, unheparinized, and TXA groups. The TXA group did have lower EBL, but it was not statistically significant, suggesting that a higher patient number might reveal a significant effect, warranting further study. Unlike our study, Claydon et al. [7] did not examine differences in blood transfusion rates between their groups. In our study, no patients in the TXA group required blood transfusions, while some patients in other groups did. This finding is important, as it may explain why patients in the TXA group were more likely to be discharged home without supplemental services, a topic for future research. Surprisingly, TXA patients did not require transfusions, even though none of them underwent a standalone anterior approach, pointing to a potential area for further investigation.

Determining the significance of differences found in our study is challenging due to numerous extraneous variables inherent in ALIF procedures, such as surgeon experience, the type of surgeon performing the exposure and fusion, the rate of vessel injuries, the subjectivity in reporting EBL, and the spinal approach. A study by Vint et al. [9] proposed a venous thromboembolism (VTE) prophylaxis regimen for ALIF procedures using heparin, tinzaparin, acetylsalicylic acid, and lansoprazole. Similar to heparin, tinzaparin inactivates factor Xa by binding to antithrombin III to act as an anticoagulant [10]. Lansoprazole is a proton-pump inhibitor that reduces the risk of gastric bleeding associated with non-vitamin K antagonist anticoagulants [11]. In their cohort of 200 patients, the use of preoperative heparin and tinzaparin, combined with postoperative heparin, tinzaparin, acetylsalicylic acid, and lansoprazole, resulted in no symptomatic VTE occurrences, wound hematomas, or need for transfusion [9]. Their promising outcomes suggest potential for further investigation into optimized coagulation intervention protocols.

The incorporation of the antifibrinolytic drug TXA in our analysis sheds light on the use of a hemostatic agent that has limited reporting in the literature for ALIF procedures. TXA controls bleeding by binding to plasminogen, thereby preventing plasmin from binding to fibrin and breaking down blood clots [12]. Its use in controlling bleeding is well documented in fields such as obstetrics and trauma surgery, including the CRASH-2 randomized control trial [13,14]. TXA has been proven safe across various surgical procedures, with severe adverse events being rare in clinical trials [13,15]. It has been increasingly used in spine surgeries to manage large-volume blood loss, reduce blood transfusions, and lower complication rates [16]. Our study's lack of association between TXA and significant surgical complications, outcomes, or operative measures reinforces the drug's safe use in optimizing ALIF outcomes. Our findings indicate that the TXA group exhibited a trend toward lower blood loss, no need for blood transfusions, a higher likelihood of being discharged home without supplemental services, and the best neurologic outcomes. These results could inform surgeons' decision-making and lead to more patient-centered treatment plans during ALIF procedures.

No other study has analyzed the association between preoperative and postoperative motor function changes and anticoagulation strategy. Although not statistically significant, the observed trend toward improved motor function in the TXA group may suggest a neuroprotective role for TXA. This role has been primarily studied in traumatic brain injury (TBI) settings, with the CRASH-3 trial showing that TXA reduces early death due to increased intracranial bleeding [17]. In mouse models, TXA reduced neuroinflammation by preventing the expression of Toll-like receptor 4 and tumor necrosis factor at spinal injury sites, with improved motor function post-TBI observed in mice receiving TXA [18,19]. In orthopedic surgery patients, TXA was associated with decreased levels of C-reactive protein and interleukin-6, suggesting anti-inflammatory effects in humans [20,21]. Further analysis is needed to enhance the statistical power of these findings and determine if TXA should be administered to all ALIF patients. The low incidence of PEs and DVTs in our cohort raises questions about the association between ALIF procedures and thrombotic events. Variability in thrombotic event rates following anterior spinal approaches in the literature underscores the need for a large-scale, multi-institutional analysis to comprehensively understand this association [22-25].

This study has several limitations that should be considered. The study was conducted at a single academic center, limiting generalizability, and its retrospective design introduces selection bias and confounding variables. The lack of randomization and surgeon-driven intervention choices further weaken causal interpretations. Additionally, the small and imbalanced sample sizes across groups may reduce statistical power to detect meaningful differences.

## Conclusions

This study explored the role of perioperative coagulative intervention strategies in ALIF procedures, focusing on their impact on complication rates, blood loss, and postoperative outcomes, with findings suggesting that the use of TXA may be associated with decreased blood transfusion requirements and improved neurologic outcomes. Given the variability in coagulation management practices and significant confounding variables, the results of this study underscore the need for larger, prospective trials to validate these findings and develop standardized coagulation intervention protocols to optimize patient outcomes in ALIF procedures. Additionally, investigations into patient selection criteria, optimal dosing strategies, and long-term effects of coagulation intervention could refine practices. Notably, there were very few thrombotic events in the cohort presented. A multi-institutional study should be performed to evaluate the association between coagulative intervention and the risk of thrombotic events in ALIF procedures.

## Appendices

**Table 8:** Summary of Statistical Models This table presents validating statistical summaries for the logistic regression analyses evaluating the association between different coagulative interventions and various complications presented in Table 2. ^1^Defined as PE and DVT CVA: cerebral vascular accident; DVT: deep vein thrombosis; PE: pulmonary embolism; SSI: surgical site infection; SOB: shortness of breath; UTI: urinary tract infection

Complication	χ^2^ (Model)	p (Model)	χ^2^ (Hosmer-Lemeshow)	p (Hosmer-Lemeshow)
DVT	12.324	0.055	0.000	1.000
Leg swelling	10.734	0.097	6.308	0.613
Calf pain	11.284	0.080	6.077	0.639
SOB	2.157	0.905	7.562	0.477
PE	5.351	0.500	0.082	1.000
Thrombotic events^1^	8.228	0.222	4.406	0.819
Vessel injuries	2.299	0.890	1.223	0.996
UTI	7.908	0.245	1.600	0.991
SSI	3.673	0.721	0.361	1.000
Hematoma	2.714	0.844	1.430	0.994
CVA	3.126	0.793	7.619	0.472
Durotomy	5.893	0.435	0.004	1.000
Ileus	9.579	0.144	8.074	0.426
